# Proteomic Study of *Entamoeba histolytica* Trophozoites, Cysts, and Cyst-Like Structures

**DOI:** 10.1371/journal.pone.0156018

**Published:** 2016-05-26

**Authors:** Milka Luna-Nácar, José Navarrete-Perea, Bárbara Moguel, Raúl J. Bobes, Juan P. Laclette, Julio C. Carrero

**Affiliations:** Department of Immunology, Instituto de Investigaciones Biomédicas, Tercer Circuito Exterior s/n, Ciudad Universitaria, Universidad Nacional Autónoma de México, 04510, México D.F., México; Centro de Investigacion y de Estudios Avanzados del Instituto Politecnico Nacional, MEXICO

## Abstract

The cyst stage of *Entamoeba histolytica* is a promising therapeutic target against human amoebiasis. Our research team previously reported the production *in vitro* of Cyst-Like Structures (CLS) sharing structural features with cysts, including rounded shape, size reduction, multinucleation, and the formation of a chitin wall coupled to the overexpression of glucosamine 6-phosphate isomerase, the rate-limiting enzyme of the chitin synthesis pathway. A proteomic study of *E*. *histolytica* trophozoites, cysts, and *in vitro*-produced CLS is reported herein to determine the nature of CLS, widen our knowledge on the cyst stage, and identify possible proteins and pathways involved in the encystment process. Total protein extracts were obtained from *E*. *histolytica* trophozoites, CLS, and partially purified cysts recovered from the feces of amoebic human patients; extracts were trypsin-digested and analyzed by LC-MS/MS. In total, 1029 proteins were identified in trophozoites, 550 in CLS, and 411 in cysts, with 539, 299, and 84 proteins unique to each sample, respectively, and only 74 proteins shared by all three stages. About 70% of CLS proteins were shared with trophozoites, even though differences were observed in the relative protein abundance. While trophozoites showed a greater abundance of proteins associated to a metabolically active cell, CLS showed higher expression of proteins related to proteolysis, redox homeostasis, and stress response. In addition, the expression of genes encoding for the cyst wall proteins Jessie and Jacob was detected by RT-PCR and the Jacob protein identified by Western blotting and immunofluorescence in CLS. However, the proteomic profile of cysts as determined by LC-MS/MS was very dissimilar to that of trophozoites and CLS, with almost 40% of hypothetical proteins. Our global results suggest that CLS are more alike to trophozoites than to cysts, and they could be generated as a rapid survival response of trophozoites to a stressful condition, which allows the parasite to survive temporarily inside a chitin-like resistant cover containing Jacob protein. Our findings lead us to suggest that encystment and CLS formation could be distinct stress responses. In addition, we show that cysts express a high number of genes with unknown function, including four new, highly antigenic, possibly membrane-located proteins that could be targets of therapeutic and diagnostic usefulness.

## Introduction

Amoebiasis, a disease caused by the protozoan parasite *Entamoeba histolytica*, is the second cause of death due to intestinal parasitic infections [1, 2]. The Global Burden of Diseases, Injuries, and Risk Factors Study estimated in 55,500 the number of deaths due to amoebiasis worldwide in 2010 [3]. Amoebiasis is acquired by ingesting water or food contaminated with *E*. *histolytica* cysts. This latent stage, whose chitin wall allows for its survival outside the host, is responsible for disseminating the parasite from one host to another, spreading the disease. Excystation occurs once the cyst reaches the small intestine, particularly the ileum, releasing trophozoites, the parasite stage that proliferates in the human large intestine and is responsible for the symptoms of amoebiasis [1, 4]. Most studies on *E*. *histolytica* infection have focused on the trophozoite stage, which is relatively easy to grow in axenic culture media. Unfortunately, information on the cyst stage is very scarce due to its inability to replicate and the lack of reproducible methods for promoting trophozoite encystment and cyst excystation *in vitro*.

Recently, omics technologies have provided large amounts of information on genome expression. While over 15 transcriptome studies have been performed on *E*. *histolytica* trophozoites under different conditions, to our knowledge only one study has been done on the cyst stage [5–8]. The same goes for proteomic studies on amoebiasis. Most works have focused on analyzing the expression profiles of trophozoites in different cell organelles under various conditions. By two-dimensional electrophoresis followed by MS, it has been analyzed whole extracts, and both soluble and insoluble proteins (400–1500 spots) [9, 10], several membrane, cytoskeletal, and signaling-associated proteins as well as metabolic and ribosomal proteins attached to the Gal/GalNac lectin [11]. Other studies have included phagosome composition and proteins associated to the phagocytosis process [12–16], the cell surface proteome that identified 12 protein families [17], the pre-mRNA splicing complexes that identified 36 proteins as involved, and other 50 proteins as possibly involved in co-transcriptional processes [18], 33 proteins modulated in cells overexpressing the EhPC4 transcription factor [19] and a pull-down assay that identified two endoribonucleases interacting with the EhCAF1 deadenylase [20]. In a more recent study, the components of trophozoite ER and Golgi apparatus were determined. About 1,500 proteins were grouped into two main classes, traffic machinery and GTPases, and about 100 markers were identified in both compartments [21]. Additionally, a comparison of the *E*. *histolytica* proteome with that of the related non-pathogen amoeba *E*. *dispar* has allowed the identification of peroxiredoxin and alcohol dehydrogenase 3 as proteins associated to virulence and pathogenicity [22,23].

In contrast, only one proteomic study on the cyst stage of *E*. *histolytica* has been published to date. In that work, cysts were partially purified from five stool samples from patients infected with *E*. *histolytica* and each sample was analyzed by LC-MS/MS. In total, 417 different proteins were identified in the five samples, 95 of which were classified as stage-specific [24]. Although that study provided valuable information about the composition of the cyst, there are still no studies focusing on the process of encystment in *E*. *histolytica*, which could have a high impact on the disease control. Encystment of the reptilian parasite *E*. *invadens* has been used as a model for the process in *E*. *histolytica*. However, unlike *E*. *invadens* that encyst *in vitro* relatively easy [25–28], the stimuli capable of inducing differentiation in *E*. *histolytica* remain unknown, suggesting that the process is more complex than in the related species. Another approach to studying the encystment process in *E*. *histolytica* arose with the description of cyst-like structures (CLS) obtained from trophozoites cultured under different conditions. Thus, detergent-resistant CLS have been obtained *in vitro* by incubating trophozoites with a mixture of Mg^2+^, Mn^2+^, and Co^2+^ ions [29] or with enterobacteria at a high CO_2_ tension in the presence of histamine [30]. Moreover, chitin-like material was detected by HPLC in CLS obtained with the mixture of Mg^2+^, Mn^2+^, and Co^2+^ ions, suggesting that this treatment stimulates *E*. *histolytica* pathways related with chitin-like biosynthesis [31]. CLS described in a study by our research group were obtained under treatment of axenic trophozoites with 4mM hydrogen peroxide plus traces of metallic cations for 4 h [32]. These CLS showed features closely resembling genuine cysts, including rounded shape, size reduction, refringence, multinucleation (mainly 2 or 3 nuclei, and occasionally 4), and overexpression of glucosamine 6-phosphate isomerase, the rate-limiting enzyme in the chitin synthesis pathway [32]. In a subsequent study, CLS formation by hydrogen peroxide was inhibited in trophozoites in which glucosamine 6-phosphate isomerase expression was silenced by siRNA [33].

This study aimed to determine whether hydrogen peroxide-obtained CLS constitute an intermediate stage in the transformation of *E*. *histolytica* trophozoites into cysts, by performing a global comparison of the proteomes of *E*. *histolytica* trophozoites, cysts, and CLS obtained by LC-MS/MS. In addition to shed light on the proteomic nature of *E*. *histolytica* CLS obtained *in vitro*, this study provides new data on cyst composition, including several previously unpublished potential therapeutic and diagnostic targets.

## Material and Methods

### *Entamoeba histolytica* cultures

*E*. *histolytica* strain HM1: IMSS trophozoites were maintained in TYI-S-33 medium [34] supplemented with 15% adult bovine serum (Microlab), 3% vitamins (SAFC, Biosciences), and 1% antibiotic/antimycotic (Gibco) at 37°C under axenic conditions for 72 h.

### Cyst-like structures (CLS) induction

CLS induction was performed as previously reported [32]. Briefly, trophozoites were cultured in 50-ml bottles containing TYI-S-33 medium (approximately 5 × 10^6^ trophozoites per bottle) for 72 h and treated for 4 h with hydrogen peroxide 4 mM, 0.02 ppm of each Co, Cd, Cu, Ni, and Zn cations, and 0.01 ppm of Fe (Merck). After treatment, CLS were extensively washed with phosphate-buffered saline (PBS), pH 7.4, at 4°C, centrifuged at 150 × *g* for 5 min, resuspended in sarkosyl 0.1% in PBS, and allowed to sit for 20 min at room temperature. After three washes, the pellets of detergent-resistant CLS were processed to obtain protein extracts. In addition, aliquots of unstained and calcofluor white-stained CLS were analyzed under light and UV microscope, respectively, and used as fresh as possible in immunofluoresce experiments.

### Human stool samples

Human stool samples were provided by Laboratories CARPERMOR (Mexico City) following all applicable ethical protocols, including patient signed consent for sample donation. Approximately 80 samples, coprology-positive for complex *E*. *histolytica/E*.*dispar*, and as fresh as possible were processed for DNA isolation following all security measures for a biological sample level 2, as described below.

### DNA purification from human stool

The QIAamp® DNA Stool Kit (Qiagen) was used to purify DNA from human stool samples according to the supplier's directions. Briefly, 200 mg of each stool sample were weighed into a 2-ml microcentrifuge tube, added with 1.4 ml of ASL buffer and homogenized by vortexing. The mixture was heated for 5 min at 70°C and then homogenized and centrifuged at maximum speed for 1 min. The supernatant (approximately 1.2 ml) was placed in a new 2-ml tube and an Inhibitex tablet was added; the mixture was homogenized until dissolved and incubated for 1 min at room temperature. The tube with the mixture was centrifuged at full speed for 3 min and the supernatant (200 μl) was placed in a 1.5-ml tube containing 15 μl of proteinase K and 200 μl of AL buffer. The solution was mixed for 15 s until homogenized and incubated at 70°C for 10 min. After incubation, 200 μl of 96–100% ethanol was added, and the mixture was placed in an affinity column. The column was centrifuged at maximum speed for 1 min, washed twice with 500 μl of each AW1 and AW2 buffers, and centrifuged at maximum speed for 3 min. The column was transferred to a 1.5-ml tube, and the DNA was eluted from the column with 200 μl of AE buffer, incubated for 1 min at room temperature and centrifuged at maximum speed for 1 min. The obtained DNA was stored at –20°C until used.

### *E*. *histolytica* and *E*. *dispar* differential detection by PCR

Since *E*. *dispar* cysts are not visually distinguishable from *E*. *histolytica* cysts, a PCR assay was performed to identify both species in DNA from the coprology-positive feces obtained as mentioned above. A common forward primer EntaF 5'-ATG CAC GAG AGC GAA AGC AT-3’ and two species specific reverse primers EhR 5'- GAT CTA GAA ACA ATG CTT CTC T-3’ and EdR, 5'-CAC CAC TTA CTA TCC CTA CC-3’, were used for *E*. *histolytica* and *E*. *dispar* identification, respectively [35]. PCR reactions were performed on 100 ng of purified DNA using a HotStar Taq plus Master Mix Kit (Qiagen), according to the manufacturer’s directions. Reactions were run as follows: 94°C for 7 min; 35 cycles of 94°C for 90 s, annealing for 1 min; 72°C for 1 min; and final incubation at 72°C for 7 min. Annealing temperature was set to 56°C for *E*. *histolytica* and to 58°C for *E*. *dispar* specific reverse primers, respectively. Amplicons were separated in 1%-agarose gels after staining with ethidium bromide, and visualized under UV light.

### Cyst isolation

Cysts were partially isolated from 10 stool samples positive for *E*. *histolytica* by PCR, as reported elsewhere [36]. Briefly, approximately 7 g of each stool sample were homogenized with 40 ml of PBS. Large particles were removed by filtering through a double-layer gauze. Filtrates were centrifuged at 150 × *g* for 7 min at 4°C; supernatants were decanted and pellets were extensively washed with PBS until supernatant was clear. Pellets were resuspended in 5 ml of distilled water. Suspensions were gently layered onto 40 ml of sucrose 1.5 M and centrifuged at 500 × *g* for 10 min at 4°C. Immediately, the cyst-containing interface was carefully removed, mixed with 20 ml of distilled water and centrifuged at 150 × *g* for 5 min at 4°C. Pellets were resuspended again in 5 ml of distilled water, layered onto 15 ml of sucrose 0.75 M, and centrifuged at 500 × *g* for 15 min at 4°C. Pellets containing cysts were stored at 4°C in PBS with sodium azide until used.

### Preparation of *E*. *histolytica* trophozoite, CLS and cyst protein extracts

To obtain trophozoite total extracts, cultures of 72 h were chilled on ice for 5 min and the cells harvested by centrifugation at 150 × *g* for 5 min. Then, trophozoites were transferred to Falcon tubes and washed five times with PBS followed by centrifugation at 150 × *g* for 5 min. Pellets from trophozoite or CLS mentioned above were washed and placed in 1 ml of PBS with protease inhibitors (Complete tablet, Sigma) on ice for 15 min. Then, samples underwent 10 liquid nitrogen/37°C freeze-thaw cycles and were centrifuged at 16,000 × *g* for 30 min at 4°C. Supernatants (total protein extracts) were transferred to clean tubes, and protein concentration was determined by the Bradford method [37]. Extracts were aliquoted and stored at –70°C until used.

Cyst protein extract was prepared from a pool of isolated cysts (about 2 × 10^5^) from 10 PCR-positive samples for *E*. *histolytica*, as follows: cysts were washed and placed in 500 μl of PBS with protease inhibitors (Complete tablet, Sigma) on ice for 15 min. Then, samples underwent 10 liquid nitrogen/37°C freeze-thaw cycles and sonication for 30 min (15 s/15 s rest), followed by centrifugation at 16,000 × *g* for 30 min at 4°C. Supernatants (total protein extracts) were transferred to clean tubes, and protein concentration was determined by the Bradford method [37]. Cyst protein extract was stored at -70°C until used.

### Sample preparation for LC-MS/MS analysis

About 40 μg of the trophozoite, CLS and cyst protein extracts were applied to 12% polyacrylamide gels and run just enough time to allow the mixture of proteins to enter the stacking gel and concentrate as a coarse band. Bands from each sample (trophozoites, cysts and CLS) were cut under sterile conditions and sent to Bioproximity Laboratories (Chantilly, VA) for LC-MS/MS analysis.

### Protein denaturation and digestion

Samples were prepared for digestion by the filter-assisted sample preparation (FASP) method [38]. Briefly, the samples were suspended in 2% SDS, 50 mM Tris-HCl, pH 7.6, 3 mM DTT, briefly sonicated, and incubated at 40°C for 20 min. Samples were clarified by centrifugation, and then supernatants were transferred onto 30-kDa MWCO Amicon columns (Millipore), centrifuged at 13,000 × *g* for 30 min and resuspended in 8 M urea, 100 mM Tris-HCl, pH 7.6. Proteins in the sample were then alkylated with 15 mM iodoacetamide. After diluting in 2M urea, proteins were digested overnight with trypsin at an enzyme: substrate ratio of 1:40 at 37°C with constant agitation. Digested peptides were collected by centrifugation.

### Peptide desalting

About 20 μg of the digested peptides were desalted using C18 STAGE stop-and-go extraction tips [39]. Briefly, a C18 STAGE tip was activated with methanol for each sample, and then conditioned with acetonitrile 60%:acetic acid 0.5%, followed by acetonitrile 2%/acetic acid 0.5%. Samples were loaded onto the tips and desalted with acetic acid 0.5%. Peptides were eluted with acetonitrile 60%:acetic acid 0.5% and lyophilized in a SpeedVac (Thermo Savant) apparatus to near dryness.

### Liquid chromatography-tandem mass spectrometry (LC-MS/MS)

Peptides were analyzed by LC-MS/MS. LC was performed on an Easy-nLC 1000 UHPLC system (Thermo Scientific, Waltham, MA). Mobile phase A was 97.5% MilliQ water, 2% acetonitrile, 0.5% acetic acid. Mobile phase B was 99.5% acetonitrile, 0.5% acetic acid. LC gradient ran from 0% B to 35% B over 210 min, then to 80% B for further 30 min. Samples were loaded directly into a C18 50 cm × 75 μm column (Thermo Easy Spray PepMap). LC was interfaced to a quadrupole-Orbitrap mass spectrometer (Q-Exactive, Thermo Fisher) via nano-electrospray ionization using a source with an integrated column heater (Thermo Easy Spray). The column was heated to 50°C. An electrospray voltage of 2.2 kV was applied. The mass spectrometer was programmed to acquire, by data-dependent acquisition, tandem mass spectra from the top 20 ions in the full scan from 400–1200 m/z. Dynamic exclusion was set to 15 s, single-charged ions were excluded, isolation width was set to 1.6 Da, full MS resolution to 70,000 and MS/MS resolution to 17,500. Normalized collision energy was set to 25, automatic gain control to 2e5, maximum fill MS to 20 ms, maximum fill MS/MS to 60 ms and the underfill ratio to 0.1%.

### Data processing and library searching

Mass spectrometer RAW data files were converted to the mz5 format using the ms convert [40]. Briefly, all searches required 10 ppm precursor mass tolerance, 0.02 Da fragment mass tolerance, strict tryptic cleavage, 0 or 1 missed cleavages, fixed modification of cysteine alkylation, variable modification of methionine oxidation, and expectation value scores of 0.01 or lower. Proteins were required to have 1 or more unique peptides across the analyzed samples with E-value scores of 0.01 or less.

### Bioinformatic analysis

Proteins identified in trophozoite, cyst, and CLS total extracts were analyzed with the BioVenn software for Venn diagram construction, STATISTICA 12 for correlation analysis, and DAVID Bioinformatics Resources 6.7 for functional classification [41, 42]. The proteins identified were also mapped with Gene Ontology terms using the Panther DB [43,44], and the overrepresentation test was performed using a *P*-value < 0.05. Those proteins that could not be annotated using Panther were annotated with Argot2 (total score > 200) [45–47]. The sub-cellular location of some proteins was predicted using CELLO v2.5 [48,49], and antigenicity was predicted using the Kolaskar and Tongaonkar antigenicity scale [50].

### *E*. *histolytica* trophozoites, CLS and cyst RNA purification

RNA was purified from trophozoites or CLS with the Direct-zol RNA MiniPrep kit (Zymo Research, Irvine, CA). Briefly, approximately 10^6^ trophozoites or CLS were added with 1 ml of Trizol and homogenized by vortexing. Subsequently, 1 ml of ethanol was added, and the homogenate was vortexed and placed on a Zymo-Spin IIC Column and centrifuged for 1 min at maximum speed. Then, the column was treated with 400 μl of RNA Pre Wash and washed with 700 μl of RNA Wash buffer, and then centrifuged for 1 min at maximum speed. Finally, the column was transferred to an Eppendorf tube and RNA was eluted with 50 μl of RNase-free water. RNA was quantified and stored at –70°C until used.

Cyst RNA was purified from the 0.25 g of sample stool with the Power Microbiome RNA isolation kit (MO BIO Laboratories, Carlsbad, CA) following the manufacturer's directions. Briefly, stool was placed into a 1-ml tube; 650 μl of PM1 were added; the tube was vortexed for 10 min and centrifuged at maximum speed for 1 min. The supernatant was transferred into a new tube, 150 μl of PM2 solution were added, and the mixture was centrifuged for 1 min. The supernatant was collected into a new tube and 650 μl of PM3 and PM4 solutions were added. Then, 650 μl of supernatant were loaded onto a Spin Filter and centrifuged for 1 min; 650 μl of PM5 Solution were added and the Spin Filter was centrifuged for 1 min. Then, 50 μl of DNase I Solution and 400 μl of PM7 Solution were added to the Spin Filter and it was centrifuged for 1 min. The column was washed with PM5 and PM4 solutions and centrifuged for 1 min after each wash. Finally, RNA was eluted with 50 μl of RNase-free water. RNA was quantified and stored at –70°C until used.

### Reverse transcriptase PCR

RT-PCR was performed using the SuperScript One-Step RT-PCR kit with Platinum Taq (Invitrogen, Carlsbad, CA) following the manufacturer’s specifications. Reactions were run using 200 ng of RNA from each sample. Samples were amplified as follows: 55°C for 30 min; 94°C for 2 min; 40 cycles of 94°C for 15 s; annealing for 30 s; 70°C for 1 min; and final incubation at 68°C for 10 min. Annealing temperature and the primers used are summarized in Table 1. Amplicons were resolved in 1% agarose gels for visualization.

**Table 1:** Oligonucleotides used for RT-PCR of *Entamoeba histolytica* cyst wall proteins.

Sequence Accession	Protein name	Primers F/R 5’ -3’	Amplified size	Annealing T ° C
XM_001913371.1	Malic enzyme, putative	ACTCGGATCCATGGCACAATTAAAAGCAGATTCCTCGAGTTTTCCAGTGACTTTGTTAA	1464	52
XM_645281.2	fructose-1,6-bisphosphate aldolase, putative	ACTCGGATCCATGGCTGCTAAGACTGTTAACATTCCTCGAGATACCATGATTTTCCTGCTGAG	990	61
XM_645264.2	glyceraldehyde-3-phosphate dehydrogenase, putative	ACTCGGATCCATGTCAATTAAGGTCGGTATTAATTCCTCGAGATGAACTTTAGAAATGATTTGGA	1000	58
XM_001914290.1	Gal/GalNAc (Fragment LC3)	TAGAAAGCTTATGTTCTAGTTTAACATGTCCATTTTTCTAGATTAACATGTTTTCTTTGTGTAAATAG	1028	59
XM_001914339.1	Peroxiredoxin	ACTCGGATCCATGTCTTGCAATCAACAAAAAGATTCCTCGAGTCTTGTTTGTTTTAATGTTGTTAA	711	58
AF401986	Chitinase Jessie 1	AAGTGGATCCATGACACTAATTATTTTCTTACCAAGCTTTTATTTATAATTTTCATAAA	273	63
AF401987	Chitinase Jessie 2	AAGTGGATCCATGATGATATTAATCATTTTACCAAGCTTTTAACAATAATCATCATTT	294	65
AF401988	Chitinase Jessie 3	AAGTGAATTCATGAAATTAGCTGTTTTAATACCAAGCTTTTATTTTGAATAATGAACT	1800	65
AF401984.1	Lectin Jacob	AAGTGGATCCATGAAAGCATTACTTGTTATCCCAAGCTTTTAATAACATGGATTGTTA	456	66
XM_652120.2	Chitin Synthase 2,putative	AAGTGGATCCATGTCAGTGAGTTTCCTTATCCCAAGCTTTTACTCTGAATGAGAAAGG	2841	68
AF082517	ADP-ribosylation factor (ARF)	GTAGGACTTGATGCGTGAAGAATTAATGA	259	51

### Western Blot against Jacob protein

A Western blot was performed to identify the Jacob protein in CLS and cysts. 15 μg of each sample were resolved in a 12% acrylamide gel and then transferred to a PVDF membrane. The membrane was blocked with milk 10% for 1 h at 37°C. Anti-Jacob 1:1000 (kindly donated by Dr. W.A. Petri Jr.) incubated overnight at 4°C and a goat HRP-conjugated anti-rabbit 1:50,000 (62–6120 ZYMED, San Francisco, CA) incubated for 2 h at 37°C, were used as primary and secondary antibodies, respectively. The Immune complex in the membrane was developed with the Super Signal West Femto Maximum Sensitivity Substrate kit (Thermo Scientific). Extract from trophozoites was used as a negative control.

### Immunofluorescence against Jacob protein

CLS and cysts were fixed with paraformaldehyde 4% for 30 min at room temperature. Then, samples were washed three times with PBS and permeabilized with Triton 1% for 1 h at 50°C. Samples were blocked with BSA 1% for 1 h, and treated with the anti-Jacob 1:200 incubated overnight at 4°C and goat FITC-conjugated anti-rabbit 1:200 (F1262, Sigma, St. Louis, MO) incubated for 2 h at 37°C, were used as primary and secondary antibodies, respectively. Samples were immediately visualized under a fluorescence microscope. Trophozoites processed in parallel were used as a negative control.

## Results

### CLS and cyst selection for the study

**Fig 1:**
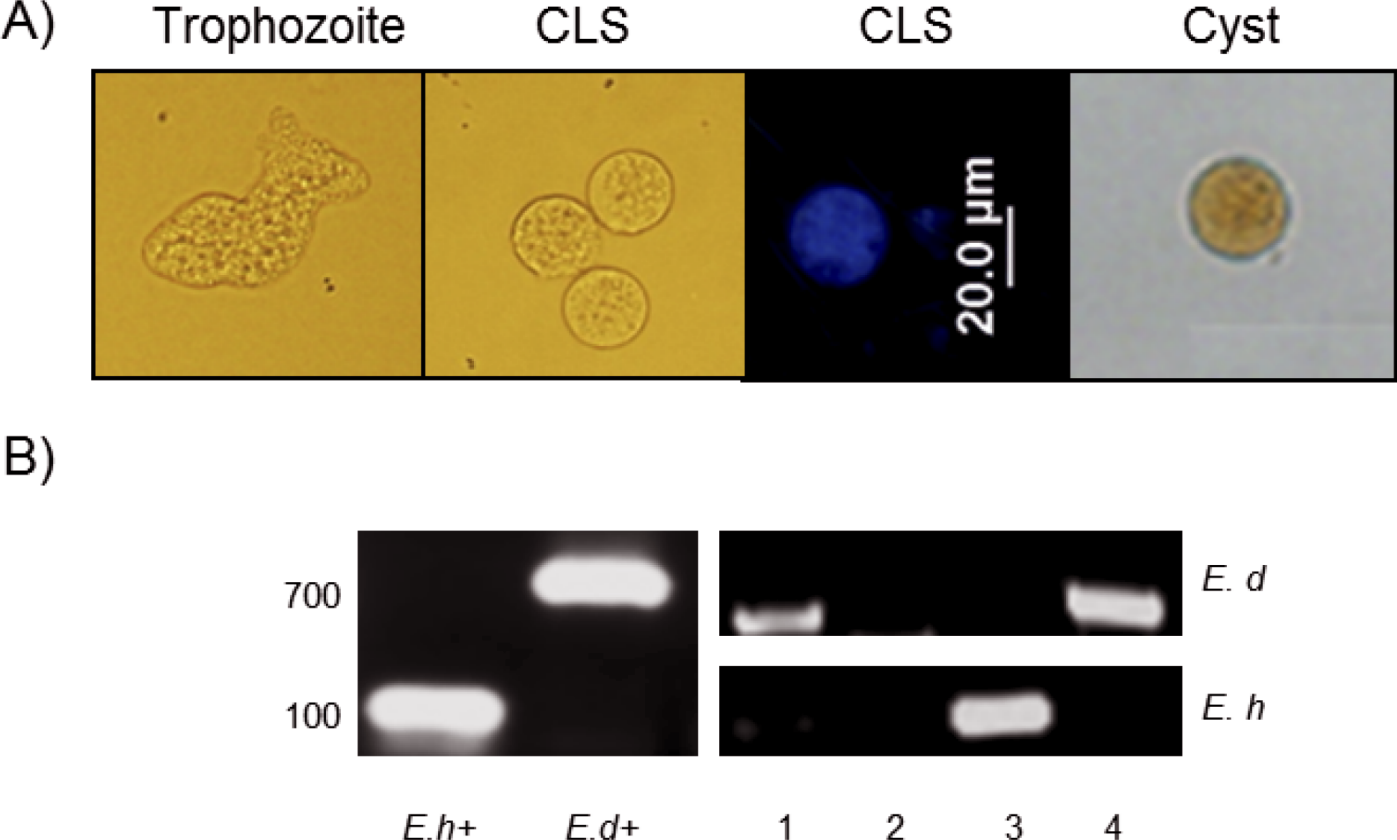
CLS induction and stool samples positive for *E*. *histolytica*. A) Samples used in this study. From left to right: *E*. *histolytica* trophozoites, CLS (induced from trophozoites after a 4 h treatment with H_2_O_2_ 4 mM), CLS stained with calcofluor white and a cysts partially purified from a fecal sample and stained with Lugol. B) Representative agarose gel of the PCR products differentiating *E*. *histolytica* from *E*. *dispar*. Samples 1 and 4 were positive to *E*. *dispar*, sample 3 to *E*. *histolytica* and sample 2 negative. *E*. *histolytica* and *E*. *dispar* trophozoite DNA were used as positive controls (Eh+ and Ed+).

CLS were obtained after a 4-h treatment with hydrogen peroxide 4 mM as previously reported [32]. After detergent resistance selection with sarkosyl 0.1% for 20 min, the morphology of the obtained structures was verified by microscopy. CLS showed a rounded shape, size reduction, and refringence that resemble cysts isolated from fecal samples (Fig 1A). In addition, the cover of CLS was strongly stained with calcofluor white (Fig 1A); previous studies using CFDA showed that CLS are still viable at this time of treatment [32]. On the other hand, 10 stool samples were selected by PCR as uniquely positive for *E*. *histolytica* (~100 bp band) from 80 putatively positive amoeba samples as determined by coproparasitoscopic analysis. The other 70 samples were excluded from this study because they were either positive by PCR for *E*. *dispar* (~700 bp band) or negative for both amoeba species. In Fig 1B, a representative agarose gel with 4 samples analyzed by PCR is shows.

### Comparison of *E*. *histolytica* trophozoites, cysts and CLS proteomic data

**Fig 2:**
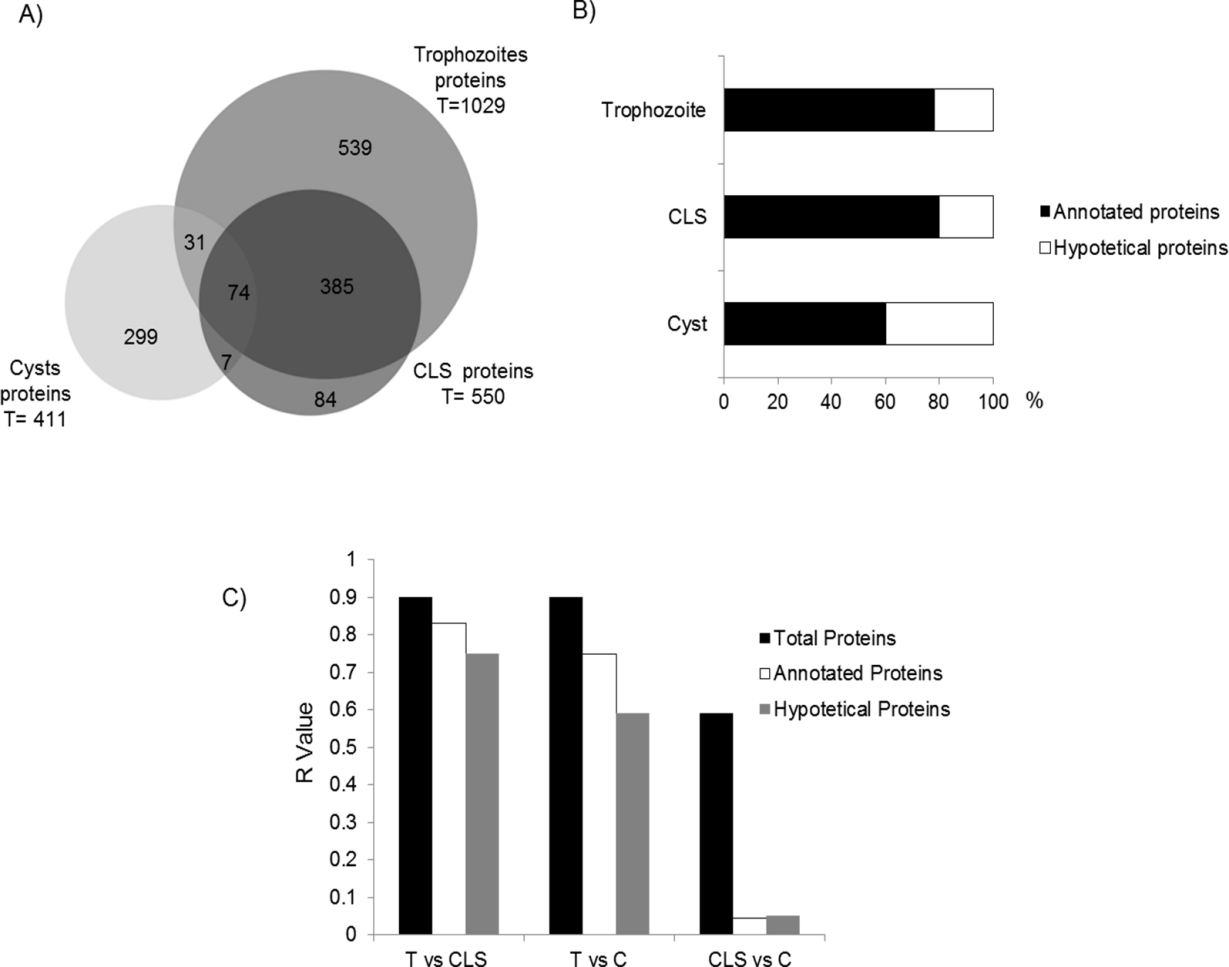
Comparison of proteomic data obtained from *E*. *histolytica* trophozoites, cysts and CLS. A) Proteome comparison using the BioVenn software. B) Percentage of annotated and hypothetical proteins identified in each sample. C) Global protein association between samples, labeled as total, annotated, or hypothetical proteins as determined by correlation test. R-values closer to 1 indicate a closer association. T: Trophozoite; CLS: cyst-like structure; C: cyst.

Raw protein extracts from trophozoites, CLS, and cysts were analyzed by mass spectrometry. In total, 1990 proteins were identified: 1029 in trophozoites, 550 in CLS, and 411 in cysts (Fig 2A). However, only 1419 *E*. *histolytica* proteins were identified as different (S1 Table). This represents about 14% of the theoretical proteome of the parasite based on 9,938 predicted genes [51, 52]. Among the 1029 proteins identified in trophozoites, 789 are annotated and 240 are hypothetical proteins (23%). Out of the 550 proteins identified in CLS, 443 are annotated and 107 are hypothetical (19%), whereas out of the 411 proteins identified in cysts 250 are annotated and 161 proteins are hypothetical (almost 40%) (Fig 2B). The number of proteins identified in trophozoites was roughly the double of those found in cysts and CLS. About 25% of all proteins identified in this study are hypothetical or have not been characterized yet (All proteomic data have been deposited in the Proteome Xchange database under Accession No. PXD004030).

A comparison of the three proteomes showed that only 74 proteins are shared among trophozoites, cysts, and CLS (Fig 2A and Table 2). Additionally, 385 proteins are shared by trophozoites and CLS, whereas 31 proteins are shared by trophozoites and cysts. Only 7 proteins are shared between cysts and CLS. The analysis also showed 539, 299, and 84 proteins unique to trophozoites, cysts, and CLS samples, respectively. Therefore, based on the numbers of different proteins either shared or unique to each sample, CLS seem more akin to trophozoites than to cysts. To verify this, a statistical correlation test comparing all proteins identified in the three samples was performed. The results showed that CLS are more closely related to trophozoites than to cysts (*R* = 0.9 vs. 0.7, respectively; Fig 2C, black bars). It is noteworthy that when proteins were grouped as annotated or hypothetical, very low association levels were found between hypothetical proteins in trophozoites and CLS with respect to cysts (0.03 and 0.05, respectively; Fig 2C, gray bars).

**Table 2:** Proteins shared among trophozoites, cysts, and CLS as identified by LC-MS/MS.

ID Accesion Uniprot	Name	No. of matched peptides /Count peptide Hits		
		Trophozoites	Cysts	CLS
S0AUW6	Actin	97/645	10/68	46/373
S0AWJ4	Actin, putative	100/645	10/68	45/358
S0AWF4	Actin	96/640	10/68	45/363
S0AWZ1	Actin	100/639	10/68	46363
S0AWJ9	Actin, putative	94/635	10/68	45/363
S0AUT4	Actin, putative	97/626	10/68	44/361
S0AUS7	Actin, putative	95/616	9/64	43/354
S0AVA7	Actin, putative	94/608	9/66	44/361
S0AWA2	Actin, putative	91/605	10/68	40/331
S0AXB5	Actin, putative	83/596	11/70	40/331
**Q24801**	**Pyruvate phosphate dikinase**	**113/587**	**2/4**	**64/285**
S0AV75	Actin, putative	92/586	10/68	45/346
Q9TYD6	Actin	96/583	10/68	43/342
S0AUR6	Actin, putative	90/583	9/64	40/332
S0AV23	Actin, putative	87/579	8/51	43/324
S0AVQ9	Actin, putative	90/568	10/68	46/337
S0AWT4	Actin, putative	74/536	8/63	34/396
B1N621	Actin, putative	79/487	9/66	38/282
S0AW09	Actin, putative	86/478	8/35	40/285
S0AV15	Actin, putative	75/444	8/38	38/280
S0AVT2	Actin, putative	66/421	7/50	35/251
**C4LVR9**	**Glyceraldehyde-3-phosphate dehydrogenase**	**71/333**	**1/2**	**30/105**
**S0AX11**	**Glyceraldehyde-3-phosphate dehydrogenase**	**69/330**	**1/2**	**30/105**
S0AYM0	Actin, putative	80/316	3/19	32/148
S0B0T0	Actin, putative	73/282	2/15	30/133
S0B1Y3	Actin	73/276	2/15	29/116
C4LU72	Myosin heavy chain	100/226	1/1	37/73
Q07569	Myosin heavy chain	83/197	1/1	31/65
B1N306	Elongation factor 2	69/195	1/1	12/28
**B1N2J0**	**Malic enzyme, putative**	**52/185**	**2/5**	**20/51**
C4LY85	Ubiquitin, putative	11/176	1/1	4/16
**G5EKL5**	**Malic enzyme**	**48/174**	**2/5**	**18/45**
**S0AW44**	**Malic enzyme, putative**	**46/172**	**2/5**	**18/48**
**S0AZU7**	**Phosphoglycerate kinase**	**33/156**	**1/1**	**15/45**
S0AWG4	Elongation factor 1-α	38/150	1/3	21/86
**C4M3S5**	**Heat shock protein 70, putative**	**45/150**	**2/5**	**19/54**
O15593	Actin	23/136	1/2	5/60
**C4LXD7**	**Fructose-1,6-bisphosphate aldolase, putative**	**45/133**	**2/3**	**16/36**
S0B0R7	Elongation factor 1-α	33/117	1/3	18/72
**C4M3Q0**	**Peroxiredoxin**	**31/98**	**1/4**	**27/124**
**C4MB38**	**Protein disulfide isomerase, putative**	**29/93**	**1/1**	**35/180**
**Q24835**	**Galactose-specific adhesin 170kD subunit**	**35/91**	**1/1**	**54/160**
**B1N3C7**	**Galactose-inhibitable lectin 170 kDa subunit, putative**	**33/90**	**1/1**	**55/166**
**B1N5A8**	**Peroxiredoxin**	**26/90**	**1/4**	**23/116**
O15729	Peptidyl-prolyl cis-trans isomerase	16/89	1/1	5/13
**C4LTM0**	**Gal/GalNAc lectin heavy subunit**	**29/81**	**1/1**	**50/153**
**B1N486**	**Heat shock protein 70, putative**	**29/69**	**2/5**	**9/28**
C4LYJ7	Galactokinase, putative	21/61	1/2	10/30
**C4M770**	**70 kDa heat shock protein, putative**	**24/59**	**2/2**	**43/177**
O15601	Elongation factor 1α	12/55	1/3	8/26
**O77164**	**70 kDa heat shock protein Hsp70-Bip**	**21/50**	**2/2**	**37/142**
C4M0F4	14-3-3 protein 3	18/47	1/4	5/11
C4LVD3	Sulfate adenylyltransferase, putative	20/43	2/2	6/11
C4M6Y2	Polyadenylate-binding protein, putative	16/28	1/1	3/5
B1N336	40S ribosomal protein S4, putative	11/27	1/1	1/2
B1N368	Putative uncharacterized protein	14/26	1/1	2/4
C4M711	Putative uncharacterized protein	9/26	1/1	2/2
C4M9P2	Filopodin, putative	11/21	1/1	6/9
**C4MBE2**	**EF-hand calcium-binding domain containing protein**	**6/17**	**1/4**	**8/72**
Q9BLE4	Small GTPase Rab11B	9/17	1/1	6/7
Q24834	Disulphide oxidoreductase	6/16	1/1	3/4
Q9BLE8	Small GTPase Rab7A	4/14	2/2	2/4
C4M6S2	60S ribosomal protein L14, putative	5/13	1/2	2/3
C4M8Z5	Putative uncharacterized protein	6/12	1/1	1/1
Q6AW60	EhRab11A protein	6/11	1/1	5/6
C4M846	26s protease regulatory subunit	6/10	1/1	2/2
C4LYV2	60S ribosomal protein L7, putative	6/9	1/1	1/2
C4LXG1	Purine nucleoside phosphorylase, putative	3/7	1/1	1/2
C4M7T9	Ubiquitin-activating enzyme, putative	4/6	1/3	2/4
B1N384	60S ribosomal protein L4, putative	2/5	1/1	1/2
C4M3X0	Calcium-transporting ATPase	3/5	2/3	2/3
Q27642	Calcium-transporting ATPase	2/4	1/1	2/3
C4LYN0	Putative uncharacterized protein	2/2	1/1	4/9
C4M0V2	Multidrug resistance protein, putative	1/1	1/1	1/3

Proteins in bold are discussed in the text

The results show that the differences in trophozoites and CLS from cysts are largely based on hypothetical proteins when the presence or absence of proteins, rather than their relative abundance, is considered. This is interesting in the sense that hypothetical proteins in *E*. *histolytica* cysts could be important for the generation and/or maintenance of this stage. When the group of 74 proteins shared by the three samples was compared on the basis of relative abundance (peptide-hit score), 24 actin entries showed the highest peptide-hit score in all three samples. It is remarkable that peptide-hits for cellular metabolism proteins like pyruvate phosphate dikinase, glyceraldehyde 3-phosphate dehydrogenase, malic enzyme, phosphoglycerate kinase, fructose 1,6-bisphosphate aldolase, and galactokinase were more than double in trophozoites than in CLS and cysts (Table 2). In contrast, peptide-hit score was higher, in some cases two or three times higher, for oxidative stress enzymes like peroxiredoxin and two HSP70 (Bip and a putative one), as well as for disulfide isomerase and EF-hand calcium-binding domain-containing proteins in CLS samples than in trophozoites and cysts. It is worth mentioning the high expression in CLS of the 170-kDa heavy subunit of galactose-inhibitable lectin, given its role in cyst wall formation in *E*. *invadens* [53]. Finally, in cysts, the abundance of malic enzyme, putative 70-kDa heat shock proteins, and a 14-3-3 protein was relatively high, in addition to actins (Table 2).

As mentioned above, hundreds of unique proteins were identified in trophozoite and cyst samples, whereas CLS shared most proteins with trophozoites. Unique proteins with a high peptide-hit score may be stage-specific and might play a functional role. A list of the 15 top highly abundant, unique proteins of each sample and their possible function is shown in Table 3. Proteins involved in DNA-binding, translation, ribosome biogenesis, and actin polymerization were identified as relatively abundant in trophozoites. Grainin, a granule calcium-binding protein probably involved in control of endocytotic pathways and granule discharge, was identified in CLS with the highest peptide-hits score. Other unique proteins involved in proton transport, proteolysis, and redox homeostasis and stress response were also identified. In contrast, cysts showed high expression levels of many putative uncharacterized proteins (8 out 15 proteins with the highest peptide-hit score) in addition to a putative nuclear transcription factor, an aminoglycoside 3’-phosphotransferase, and an alkyl sulfatase.

**Table 3:** Proteins identified as unique with highest peptide-hit score in trophozoites, CLS, and cysts.

Accession number	Protein name	No. of matched peptides/ Count peptides	Function
**Trophozoites**			
C4LV11	Putative uncharacterized protein	17/35	**unknown function**
C4LWJ6	LIM zinc finger domain containing protein	9/32	*zinc ion binding*
C4LV07	Malate dehydrogenase, putative	7/30	*oxidoreductase activity*
C4LT49	40S ribosomal protein S24, putative	11/29	**translation**
B1N3R9	60S ribosomal protein L7a, putative	10/27	**ribosome biogenesis**
C4M0A8	Putative uncharacterized protein	16/26	**unknown function**
C4M0Q2	40S ribosomal protein S8	12/24	**translation**
C4M727	60S ribosomal protein L27, putative	11/24	**translation**
C4LUC7	Non-conventional myosin IB	11/23	*ATP binding*
C4LW30	RNA modification enzymes, MiaB-family	12/23	**RNA modification**
C4LZB0	40S ribosomal protein S6	8/23	**translation**
C4M7H1	Actobindin, putative	10/23	**binds actin monomers**
C4LZ12	Tyrosyl-tRNA synthetase, putative	9/22	**tRNA aminoacylation for protein translation**
B1N2Z3	60S acidic ribosomal protein P0, putative	10/21	**ribosome biogenesis**
C4LSV0	ARP2/3 complex 20 kDa subunit, putative	10/21	**actin filament polymerization**
**CLS**			
B1N4A1	Grainin, putative	9/22	*calcium ion binding*
C4M784	Putative uncharacterized protein	10/18	*catalytic activity*
C4LTM6	Serine-threonine-isoleucine rich protein, putative	8/15	**integral component of membrane**
C4LU65	Vacuolar ATP synthase subunit H, putative	6/14	**ATP hydrolysis coupled proton transport**
B1N330	Serine-threonine-isoleucine rich protein, putative	6/11	**integral component of membrane**
C4LU31	Thioredoxin, putative	4/8	**cell redox homeostasis**
C4LUQ2	Glucosidase, putative	3/6	**carbohydrate metabolic process**
C4LTT0	Cysteine proteinase, putative	3/6	*cysteine-type peptidase activity*
C4LTV9	Uncharacterized protein	3/5	**unknown function**
C4LV34	Putative uncharacterized protein	2/5	**unknown function**
C4LT65	Heat shock protein 70, putative	1/4	**Stress response**
C4M916	Copine, putative	2/4	*calcium-dependent membrane binding proteins*
C4LX23	RhoGAP domain containing protein	2/4	**signal transduction**
C4M6M0	Putative uncharacterized protein	1/4	**small GTPase mediated signal transduction**
C4M741	Cortexillin, putative	2/4	**Cytokinesis**
**Cyst**			
C4M4Q8	Nuclear transcription factor, putative	1/29	*sequence-specific DNA binding*
C4M6B9	Putative uncharacterized protein	1/23	*RNA binding*
B1N624	Aminoglycoside 3'-phosphotransferase, putative	4/16	**response to antibiotic**
C4M8Z0	Alkyl sulfatase, putative	1/15	*alkyl sulphatase* activity
C4MBE9	Putative uncharacterized protein	2/12	**unknown function**
C4M1F3	Putative uncharacterized protein	1/11	**unknown function**
C4M977	Putative uncharacterized protein	1/11	**rRNA processing**
C4LW71	DNA mismatch repair protein PMS1, putative	1/7	**mismatch repair**
C4LX70	Putative uncharacterized protein	1/5	**double-strand break repair via non-homologous**
C4M4W5	Putative uncharacterized protein	1/5	**unknown function**
C4LYB9	Putative uncharacterized protein	1/4	**unknown function**
C4M2G7	Putative uncharacterized protein	1/4	*phosphoric ester hydrolase activity*
B1N3D1	Tyrosine-protein kinase 2, putative	1/3	*ATP binding*
B9U2P3	Organellar DNA polymerase 1	2/3	**DNA replication**
C4LV85	Centromeric protein E, putative	2/3	**kinesin-like motor protein**

Molecular function shown in italics. Biological function shown in bold. Unknown function: Unknown function in *E*. *histolytica*.

**Fig 3:**
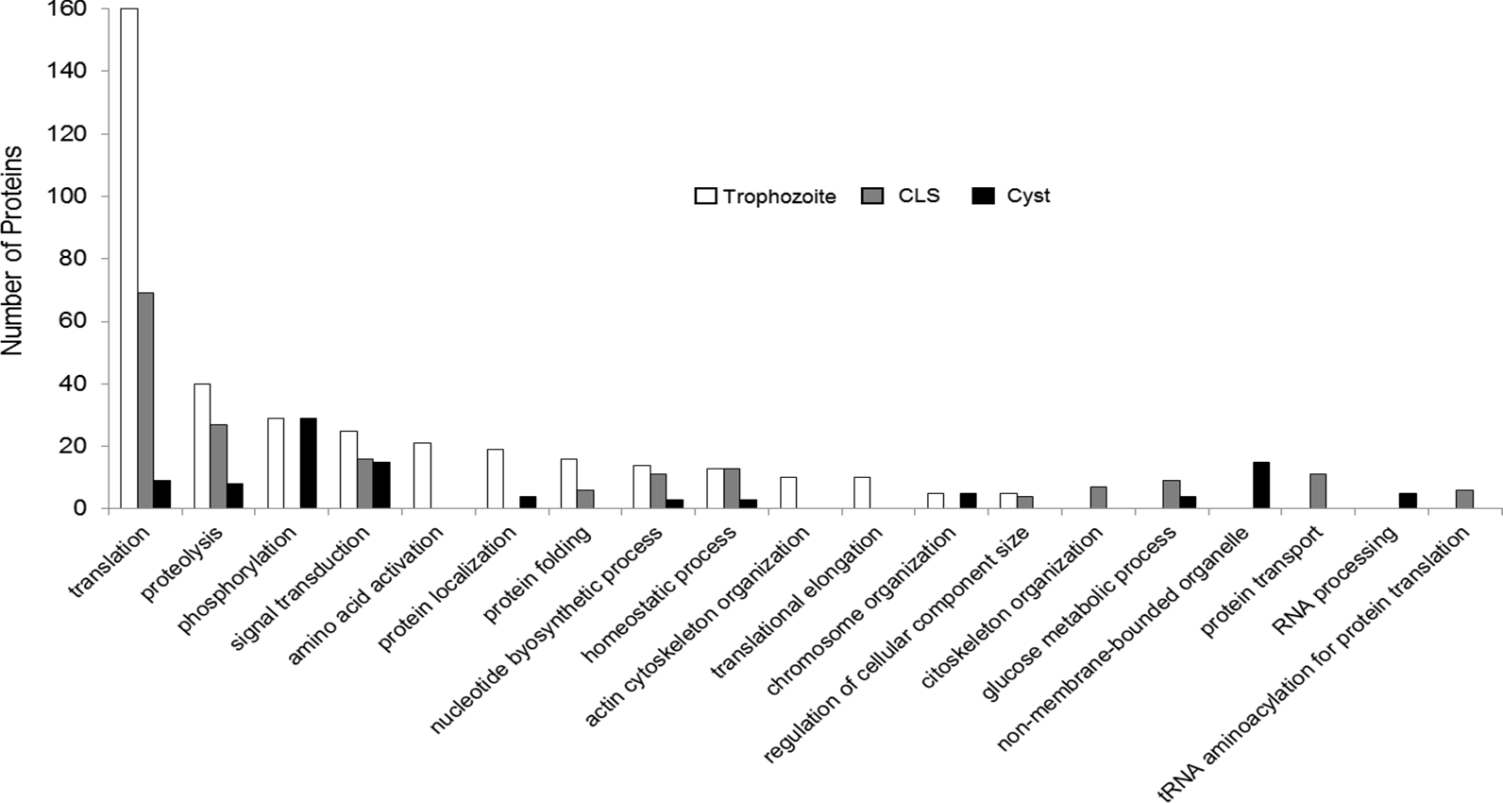
Functional classification of proteins identified in trophozoites, cysts, and CLS total extracts. The number of proteins (left y-axis) in each of 19 functional groups is shown.

Predictions of functional analysis of the annotated proteins (approximately 75% of all identified proteins) using the bioinformatics tool DAVID was performed [42]. Trophozoite, CLS, and cyst proteins were grouped into different functional groups, with trophozoites showing more functional protein groups than CLS or cysts. Trophozoite proteins were mainly grouped in clusters of translation (40%), proteolysis (15%), phosphorylation (12%), and signal transduction and amino acid activation processes (10%) (Fig 3, white bars). CLS proteins showed a pattern quite similar to trophozoites, with translation, proteolysis, and signal transduction clusters grouping over 50% of proteins, and with percentages close to those observed in trophozoites (Fig 3, gray bars). Phosphorylation, signal transduction and, notably, non-membrane bounded organelle clusters included around 60% of cyst proteins.

**Fig 4:**
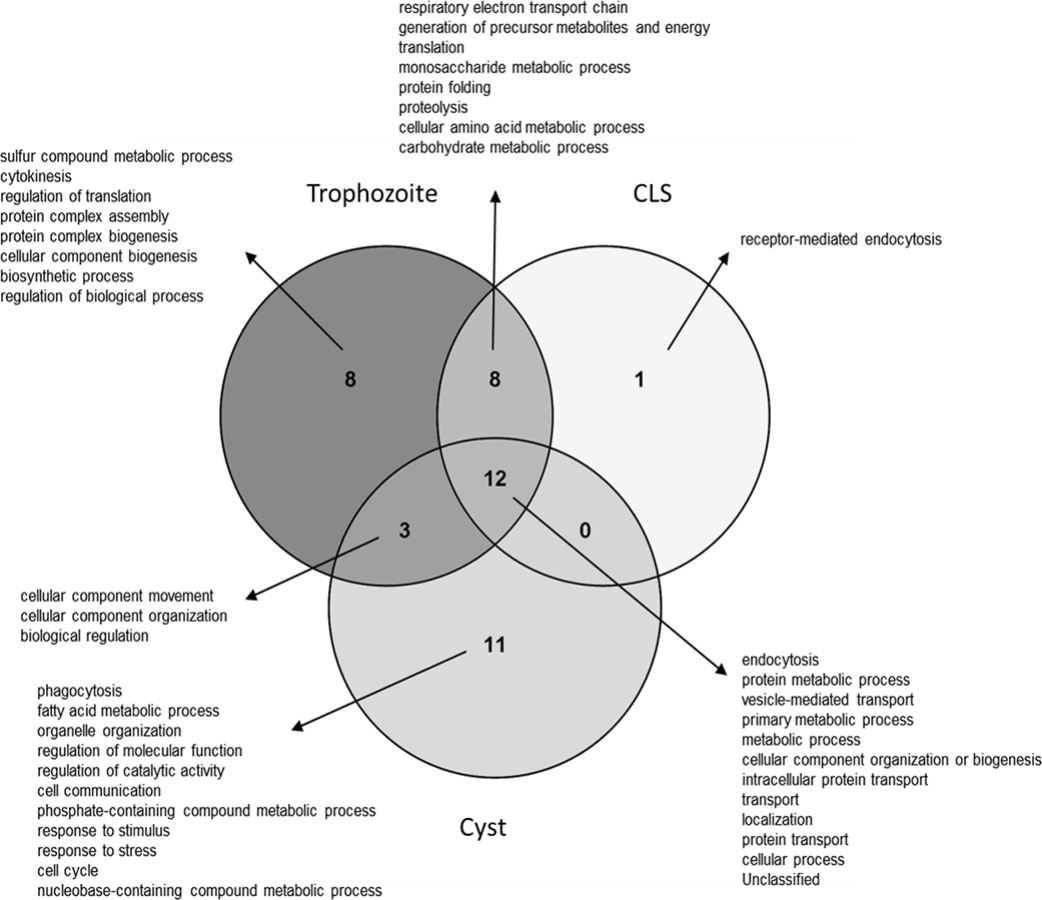
Comparison of functional categories among trophozoites, CLS and cysts. A functional comparison among the three cell types is represented in a Venn diagram. Intersections show shared functions among the analyzed samples.

The functional categories that are either shared or unique among samples are shown in Fig 4. In total, 12 categories were shared by the three samples, including metabolic, transport, and localization processes. Trophozoites and cysts shared three functional categories, while trophozoites and CLS shared eight categories. It is important to mention that cysts and CLS failed to share any functional categories. Furthermore, trophozoites, CLS, and cysts have 8, 1 and 11 unique categories.

An overrepresentation test was performed using PANTHER [43,44] to determine the biological processes that were enriched in each analyzed sample. Several processes were identified as enriched in trophozoites (*P* < 0.001): a sulfur compound metabolic process, respiratory electron transport chain, generation of precursor metabolites and energy, translation and monosaccharide metabolic processes. Confirming the high identity of CLS with trophozoites, an over-expression related to homologous processes in the former was also identified in the latter, except for the sulfur compound metabolic process. Furthermore, enrichment was also observed in other CLS processes such as proteolysis and protein folding (*P* < 0.001). Finally, the most enriched processes in cysts were phagocytosis, fatty acid metabolic processes, endocytosis, cellular component movement, and organelle organization (*P* < 0.001). It is noteworthy that translation and metabolism processes aren’t enriched in cysts. The complete enrichment analysis is shown in S2 Table.

### Validation of proteins identified in proteomic study by RT-PCR

**Fig 5:**
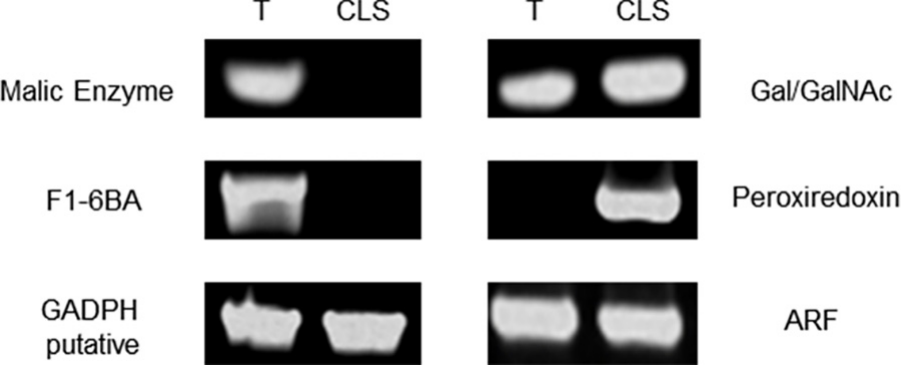
Validation of proteins identified by RT-PCR. Five proteins were selected for RT-PCR validation. Left above: malic enzyme. Left middle: F1-6BA, fructose 1,6-bisphosphate aldolase. Left below: GAPDH: glyceraldehyde 3-phosphate dehydrogenase. Right above: Gal/GalNAc lectin LC3 fragment. Right middle: peroxiredoxin. Right below: ARF, ADP-ribosylation factor, used as loading control. T: trophozoite, CLS: cyst-like structure.

Five proteins were selected to validate the proteomic findings in our study. Three proteins that were identified as less abundant in CLS with respect to trophozoites (malic enzyme, fructose 1,6-bisphosphate aldolase, and glyceraldehyde 3-phosphate dehydrogenase [GAPDH]), and two proteins more abundant in CLS than in trophozoites (peroxiredoxin, associated with oxidative stress response, and the Gal/GalNac lectin, associated to encystment). In agreement with the proteomic results, the RT-PCR showed that malic enzyme and fructose 1,6-bisphosphate aldolase were not expressed in CLS, in contrast with trophozoites where the expression was clearly detected (Fig 5). However, GAPDH showed similar expression levels in CLS and trophozoites. On the other hand, peroxiredoxin mRNA expression was observed in CLS but not in trophozoites, and the Gal/GalNAc lectin (fragment LC3 of the heavy chain) showed a 2-fold increase in CLS with respect to trophozoites (Fig 5), in accordance with the proteomic results. The ADP-ribosylation factor, used as internal control, was expressed at similar level in both samples. Unfortunately, we were unable to amplify the mRNA of none of the tested genes for validation in cyst RNA, although we amplified *E*. *histolytica* ribosomal RNA genes in that sample, suggesting that the messenger was present and intact (data not shown). The reason for the lack of amplification is unknown, but it could be related with the low concentration of *E*. *histolytica* cyst mRNA in the sample due to contamination with other components in stool, or to an arrest in RNA production because cyst is a metabolically inactive stage (latency stage). However, ribosomal RNA would be amplified because it is the most abundant RNA form in a cell. Oligonucleotides used in this study are shown in Table 1.

### Expression analysis of cyst wall proteins in CLS

Even though our results indicate that CLS resemble more trophozoites than cysts, previous studies in our group showed that CLS exhibit some morphological and structural characteristics of cysts, including multinucleation and a detergent-resistant wall susceptible to calcofluor-white staining [32]. In addition, CLS overexpress glucosamine 6-phosphate isomerase, the rate-limiting enzyme of the chitin synthesis pathway, suggesting an activation of the chitin formation route in CLS. However, except for the 170-kDa Gal-lectin subunit, other structural cyst wall proteins expected to be present in *E*. *histolytica* cysts were not identified in the current proteomic analysis, neither in CLS nor in cysts. Thus, the expression of genes encoding for some of these proteins was evaluated by semi-quantitative RT-PCR using *E*. *histolytica* specific primers for Jessie 1, 2, and 3; the Jacob protein; and chitin synthase. The ADP-ribosylation factor was included as a loading control. Primers used in this study are shown in Table 1.

**Fig 6:**
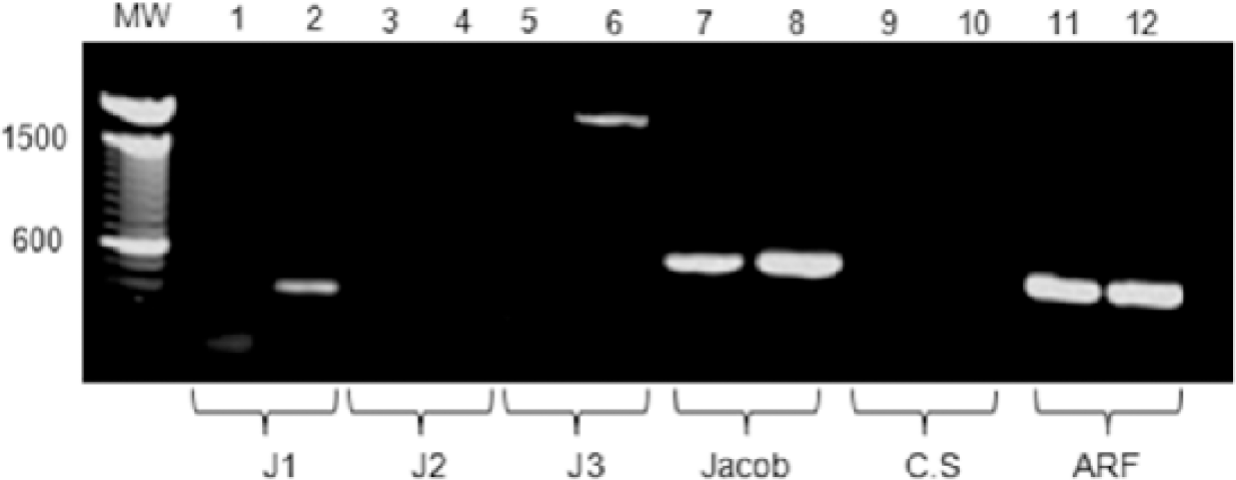
RT-PCR of transcripts encoding for cyst wall proteins in trophozoites and CLS. RNA was isolated from trophozoites and CLS samples and used to perform a RT-PCR for a number of cyst wall specific proteins: Jessie 1 (J1), Jessie 2 (J2), Jessie 3 (J3), Jacob, and chitin synthase (CS). RT-PCR of the ADP-ribosylation factor (ARF) was used as an internal control. Odd numbers: trophozoites samples; even numbers: CLS samples.

Our results showed the expression of the cyst wall proteins Jessie 1, Jessie 3, and Jacob in CLS, whereas only the Jacob protein mRNA was detected in trophozoites. Although CLS have a detergent-resistant wall suggesting the presence of chitin, no chitin synthase expression was detected in that stage (Fig 6). As mentioned above, we were unable to amplify mRNA from none of the tested genes in cyst RNA.

### Jacob protein expression in CLS

**Fig 7:**
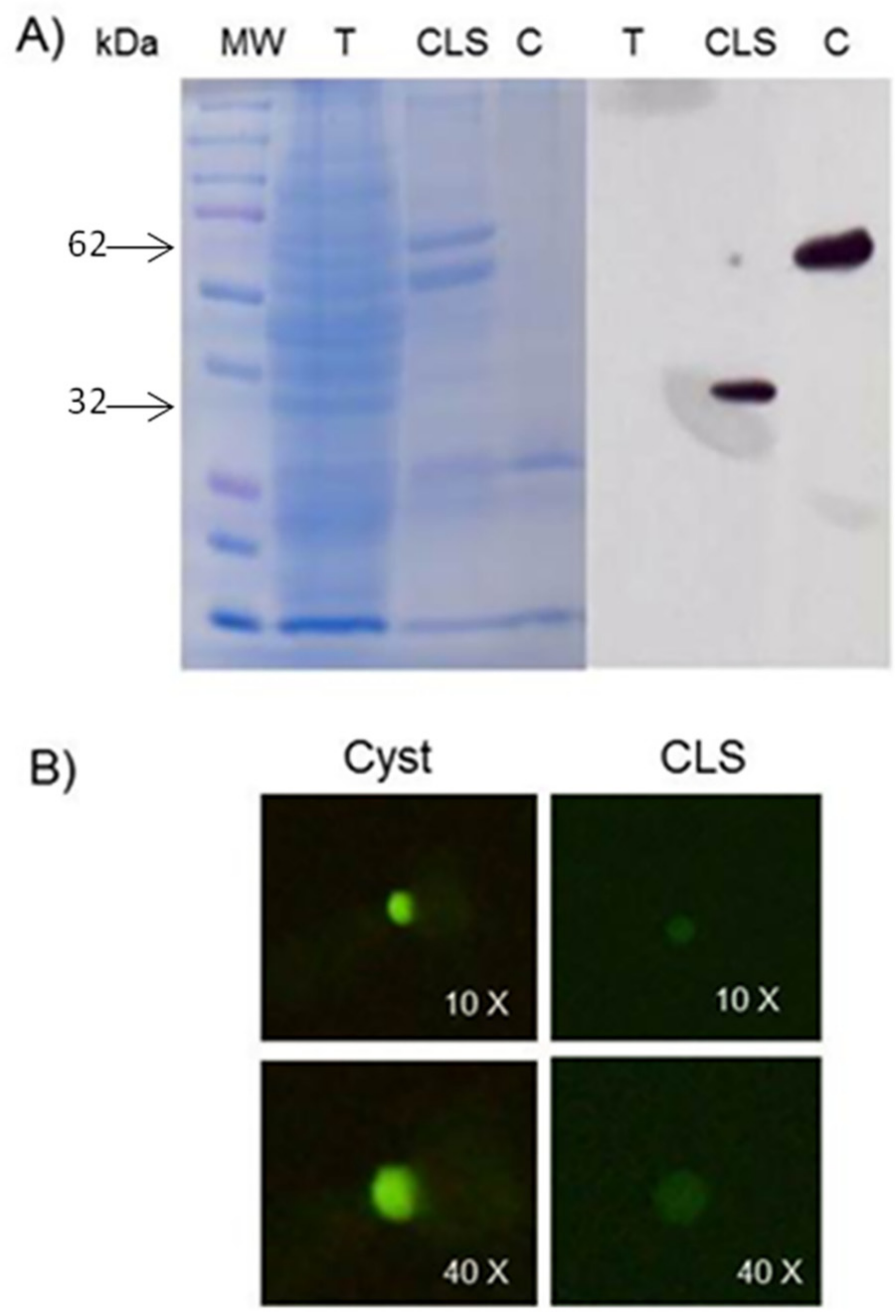
Jacob protein expression in CLS and cyst. A)15 μg of sample protein were resolved on 12% acrylamide gel (Coomassie blue-stained) and transferred to a PVDF membrane. Anti-Jacob 1:1000 and a goat HRP-conjugated anti-rabbit 1:50,000 antibodies were used. Unique bands of around 30-kDa and 62-kDa were observed in CLS and cyst samples, respectively, but not in a trophozoites sample. T: Trophozoite, CLS: Cyst-like structure, C: Cyst. B) Immunofluorescence on fixed and permeabilized cyst and CLS using anti-Jacob 1:200 and a goat FITC-conjugated anti-rabbit 1:200 antibodies.

To determine whether the Jacob protein, previously reported in *E*. *histolytica* cyst wall [24], is expressed in CLS, a Western blot of CLS extracts and immunofluorescence on CLS were performed. A clear unique band of ~32 kDa was identified in CLS extracts, whereas a unique band of ~62 kDa was identified in cysts extracts (Fig 7A). This difference could correspond to different isoforms of the Jacob protein, being previously reported as highly polymorphic in cultured amoebas and clinical isolates and showing multiple posttranslational modifications [54, 55]. Furthermore, the Jacob protein was also detected in CLS surface by immunofluorescence, although with less intensity than the signal obtained in cysts (Fig 7B). This result suggests that this cyst wall protein is expressed in CLS, but as a different isoform to that expressed in cysts.

### Analysis of cyst proteome

In 2012, Ali *et al*. published the first and to date only *E*. *histolytica* cyst proteome, using fecal samples from Indian patients [24]. To contribute to the incipient knowledge of this essential stage, our proteomic results were compared with the previously published data. While a similar number of proteins were identified in both studies (417 against 411proteins), only 16 proteins regarded as stage-specific were shared by cysts in both studies (Table 4). Therefore, the sub-cellular location of these 16 proteins using CELLO v2.5 [48,49] and their antigenicity using the Kolaskar and Tongaonkar antigenicity scale [50] were herein predicted. Our results showed 9 proteins with nuclear localization, three proteins located in the cytoplasm, two in the plasma membrane, and two with both nuclear and plasma membrane locations. Noticeably, three out the four of plasma membrane proteins presented an antigenicity score > 60%, and two of them have transmembrane regions. These proteins could be considered previously undescribed *E*. *histolytica* cyst-based therapeutic and diagnostic targets and deserve further studies (Table 4, grey labels).

**Table 4:** Proteins identified in cysts in both studies.

Gene name	ID Uniprot	Name	Sucellular localization	% Antigenicity
EH_113570	C4M9W2	Kinesin-like protein	Nuclear	43.17
**EH_056650**	**C4M9T0**	**Serine/threonine-protein phosphatase**	**Plasma membrane**	**57.17**
EH_103530	C4LVJ7	DNA primase	Nuclear	56.31
EH_110170	C4LU71	Ubiquitin carboxyl-terminal hydrolase domain containing protein	Nuclear	53.38
**EH_086140**	**C4M369**	**Ras guanine Nucleotide Exchange factor, putative**	**Plasma membrane**	**63.52**
EH_094140	C4LYK1	Rab GTPase activating protein, putative	Nuclear	63.29
EH_004750	C4M071	Signal recognition particle protein SRP54, putative	Cytoplasm	42.0
**EH_134750**	**C4M024**	**Protein tyrosine domain-containing protein**	**Plasma membrane and Nuclear**	**61.6**
EH_152390	C4LSS6	Protein kinase domain containing protein	Nuclear	63.68
EH_164910	C4M1Y4	Serine/threonine-protein kinase, putative	Nuclear	50.34
EH_010740	C4LYW1	Putative uncharacterized protein	Nuclear	56.91
EH_031640	C4MBA2	Putative uncharacterized protein	Cytoplasm	54.2
EH_123250	C4MAL5	Putative uncharacterized protein	Nuclear	33.7
**EH_146970**	**C4M8S8**	**Putative uncharacterized protein**	**Plasma membrane and Nuclear**	**63.58**
EH_148300	C4LT00	Putative uncharacterized protein	Nuclear	48.65
EH_193490	C4M4V1	Haloacid dehalogenase-like hydrolase domain-containing protein	Cytoplasm	56.19

Proteins are shown in bold with plasma membrane localization (potential therapeutic targets)

As mentioned above, about 40% of all identified cyst proteins were grouped as hypothetical proteins, roughly the double of proteins identified in the same category for trophozoites and CLS (Fig 2B). In order to further characterize those cyst proteins, all 161 hypothetical proteins were categorized using Argot2 bioinformatics tool [45–47]. Theoretical functional categories with three or more proteins, both biological and molecular, are shown in Table 5.

**Table 5:** List of functional categories, both biological and molecular, identified with Argot2 for hypothetical cyst proteins.

Biological process for hypothetical proteins	Molecular process for hypothetical proteins
Metabolic process	Hydrolase activity
Positive regulation of GTPase activity	ATP binding
Small GTPase mediated signal transduction	DNA binding
Phosphorylation	Guanyl-nucleotide exchange factor activity
Nucleosome assembly	Zinc ion binding
Protein phosphorylation	Metal ion binding
Signal transduction	Transferase activity, transferring phosphorus-containing groups
Double-strand break repair	Kinase activity
rRNA processing	Nucleic acid binding
	Nucleotide binding
	Protein serine/threonine kinase activity
	RNA binding
	Transferase activity

## Discussion

A proteomic comparison among the *E*. *histolytica* trophozoite, cyst, and CLS stages was performed for the first time in this study, employing LC-MS/MS on samples processed in parallel. In addition to contributing to the identification of new stage-specific proteins in trophozoites and cysts, which are completely differentiated parasite stages, the proteome of previously uncharacterized, detergent-resistant structures, was analyzed. These cyst-like structures were obtained *in vitro* by exposing axenic trophozoites to hydrogen peroxide 4 mM for 4 h. Similar structures have been reported by other research groups in trophozoite cultures treated with different stimuli [29–31] when attempting to promote *E*. *histolytica* encystment *in vitro*, a critical process for the continuity of the amoeba life cycle and disease transmission. The CLS obtained by our group after oxidative stress showed structural features alike to real cysts, such as multinucleation (2 and 3 nuclei), refringence, size reduction and a chitin-type cover which confers resistance to detergent concentrations that usually lyse trophozoites. In addition, CLS overexpressed the gene encoding for Gln6PI, the first enzyme in the theoretical chitin synthesis pathway in *E*. *histolytica* [32]; on the other hand, its knockdown using siRNA drastically reduced the formation of hydrogen peroxide-dependent CLS [33]. The absence of animal models for infection with *E*. *histolytica* cysts has hindered the functional comparative studies with CLS, leaving proteomic studies as a promising approach to determine the true nature of CLS and its possible relevance to understand the amoebic encystment process.

A global proteomic analysis by LC-MS/MS showed that CLS are more alike to trophozoites than to cysts. CLS shared 83.4% (459/550) of proteins with trophozoites and only 1.3% (7/550) with cysts. The main differences with trophozoites were seen on the relative abundance of proteins. These results were unexpected, since CLS obtained under exposure to hydrogen peroxide exhibited structural features resembling cysts, as mentioned above [32,33]. Even though we were unable to identify chitin-associated proteins neither in CLS nor in cysts by LC-MS/MS, we detected the presence of the cyst wall protein Jacob by WB in extracts from both samples but not in trophozoites, demonstrating that at least this protein is present in CLS. The reason behind the difference in size between Jacob proteins identified by WB in cysts and CLS is unknown, but it could be due to variants differentially expressed between the two samples, as it is well known that *E*. *histolytica* has multiple genes encoding for Jacob isoforms [54, 55]. Furthermore, the expression of mRNA encoding for the cyst wall proteins Jessie 1, Jessie 3, and Jacob, but not Jessie 2 nor chitin synthase, were identified by RT-PCR in CLS, suggesting that at least the mRNA encoding for two Jessie and the Jacob proteins were present. Thus, the difficulty in detecting cyst wall proteins in CLS by proteomic analysis might be related to issues in solubilizing chitin covers in the extracts. The previous proteomic study of *E*. *histolytica* cyst detected those proteins after analyzing five individual samples, with a very low peptide-hit score for each protein, supporting the scarcity of chitin wall-associated proteins in the extracts [24]. By contrast, in our study we evaluated a pool of cyst samples, and this may have reduced the possibility of detecting cyst wall proteins. Further studies regarding the protein composition of *E*. *histolytica* cyst and CLS walls should be conducted to confirm the presence of those proteins in cysts and to determine whether they are also present in CLS.

Proteome analysis showed that the most abundant CLS proteins include those involved in proton transport, proteolysis, redox homeostasis, and stress response. In addition, proteins involved in glycolysis and metabolism in general were found downregulated with respect to trophozoites, suggesting a protective response against oxidative shock caused by exposure to hydrogen peroxide. Noticeably, a previous report by Tomoyoshi’s group showed that oxidative stress inhibits glycolysis while increasing glycerol production, with a concomitant activation of the chitin biosynthesis pathway [56]. The results herein reported, along with the previous report on a chitin-like cover in CLS, suggest that these structures could be generated in trophozoites as a rapid survival response to a stressful condition, which allows the parasite to temporarily survive inside a chitin-like resistant cover while the insult passes. Then, we speculate that CLS could revert to trophozoites if favorable conditions are met (our unpublished observations) or under adequate (unknown) stimuli they could follow their differentiation into real cysts, a process that could involve several non-described stages and takes between 24–72 h as it has been described in *E*. *invadens* and *Giardia* sp. [28,57]. Therefore, encystment could take a long time for a protozoan to respond against environmental stress that can kill it within a few hours or even minutes. Other possibility is that CLS will not proceed to cysts under any circumstance, and both structures are part of different processes. In this regard, structures called "pseudocysts" have been reported to form under acute stress conditions, like exposition to organic solvents, in the free-living amoeba *Acanthamoeba*, responsible for keratitis in humans [58, 59]. These structures, which were formed quickly under stress, are partially resistant thanks to a simple coat formed by some of the cyst wall proteins, and are reversible to trophozoite under suitable conditions. Therefore, the authors of that work suggest that encystation and pseudocyst formation are distinct responses to stress. Although the CLS herein studied share some characteristics with *Acanthamoeba* pseudocysts, a similar pseudocyst development has not been reported so far in *E*. *histolytica*; it would be interesting to evaluate this possibility. Further structural, functional, and biochemical characterization studies on CLS are being carried out in our laboratory to confirm this possibility.

On the other hand, the proteomic study herein reported has contributed to the knowledge on the protein profile of the differentiation stages of *E*. *histolytica*, in particular the cyst stage, for which there is only one previous report [24]. Thus, the trophozoite proteome described here, equivalent to about 12% of the open reading frames predicted in the available parasite genome [51, 52], reassert it as a highly metabolically active stage, mainly expressing proteins associated to glycolysis/glycogen metabolism and cytoskeleton function, as described elsewhere [10]. Based on peptide-hit scores, actin showed the highest relative abundance, followed by proteins associated to proteolysis, phosphorylation, signal transduction, and translation, this latter representing approximately 40% of total proteins. Furthermore, metabolic enzymes such as fructose 1,6-bisphosphate aldolase, NADP-dependent alcohol-dehydrogenase-1, and phosphoglycerate kinase were also identified with high peptide-hit scores. These findings are in agreement with a previous study, where actin and these enzymes were identified as the most prominent spots in solubilized proteins from trophozoite extracts resolved by 2-D SDS [9]. In addition, we also detected proteins that have previously been associated with the virulence and pathogenicity of *E*. *histolytica* such as peroxiredoxin and alcohol dehydrogenase 3 [22,23]. It is noteworthy that 23% of the total number of proteins identified in trophozoites are still uncharacterized, and thus they represent an opportunity for future studies on new therapeutic or diagnostic targets in this parasite stage.

With respect to the cyst, herein we report 411 proteins, a number quite similar to the 417 identified in the only proteomic study of *E*. *histolytica* cysts previously reported [24]. The number of cyst proteins identified in both studies was low, probably due to a masking by human and bacterial contaminants, which counted for about 90% of proteins in the samples (data not shown). In total, 299 cyst stage-specific proteins not shared neither with trophozoites nor with CLS were identified in our study. In comparison, 195 proteins were already reported as cyst-specific, of which only 16 were shared by samples in both studies. This adds 283 new proteins to the previously identified set in cysts, contributing to the knowledge of this essential stage of the *E*. *histolytica* life cycle. Moreover, the 16 proteins identified in *E*. *histolytica* cysts isolated from patients from different geographical regions (India and Mexico) should be further explored as targets for cyst-based amoebiasis diagnostic tests; particularly, four of them were theoretically located at the plasma membrane by analysis *in silico* (Table 4). Furthermore, at least seven hypothetical and seven annotated proteins identified in cysts with the highest peptide-hit scores are also probable therapeutic targets for future studies (Table 3). Along with proteins involved in phosphorylation and signal transduction, cyst proteome showed a lower number of proteins in all functional groups (Fig 3), suggesting a general decrease in the metabolic activity consistent with what is known for latent life-cycle stages. It is noteworthy that 40% of the cyst proteome herein reported are hypothetical proteins, suggesting that our knowledge on the biology of *E*. *histolytica* cyst is still incipient and that the uncharacterized proteins, some of which showed a very high relative abundance, could play an important role in cyst formation and maintenance.

Finally, further structural, functional, and biochemical characterization studies on CLS and cysts should be carried out to determine the real nature of the former and to determine the role of the highly expressed but yet uncharacterized proteins identified in the latter.

## Supporting Information

S1 Table Total proteins identified in *E*. *histolytica* trophozoites, CLS, and cysts. (XLS)

S2 Table Overrepresentation test of *E*. *histolytica* trophozoites, CLS, and cyst proteins. (XLS)

## References

[1] Stanley SL Jr. Amoebiasis. Lancet. 2003; 361(9362):1025–34. . 12660071 10.1016/S0140-6736(03)12830-9

[2] Ali IK. Intestinal Amebae. Clin Lab Med. 2015; 35(2):393–422. 10.1016/j.cll.2015.02.009 . 26004649

[3] Lozano R, Naghavi M, Foreman K, Lim S, Shibuya K, Aboyans V, et al Global and regional mortality from 235 causes of death for 20 age groups in 1990 and 2010: a systematic analysis for the Global Burden of Disease Study 2010. Lancet 2012; 380(9859):2095–128. 10.1016/S0140-6736(12)61728-0 . 23245604 PMC10790329

[4] Ximenéz C, Morán P, Rojas L, Valadez A, Gómez A. Reassessment of the epidemiology of amebiasis: State of the art. Infect Genet Evol. 2009; 9(6):1023–32. 10.1016/j.meegid.2009.06.008 . 19540361

[5] MacFarlane RC, Shah PH, Singh U. Transcriptional profiling of *Entamoeba histolytica* trophozoites. Int J Parasitol 2005; 35 (5): 533–42. . 15826645 10.1016/j.ijpara.2005.02.006

[6] MacFarlane RC, Bhattacharya D, Singh U. Genomic DNA microarrays for *Entamoeba histolytica*: applications for use in expression profiling and strain genotyping. Exp Parasitol. 2005; 110 (3): 196–202. . 15955312 10.1016/j.exppara.2005.03.006

[7] MacFarlane RC, Singh U. Identification of differentially expressed genes in virulent and nonvirulent *Entamoeba* species: potential implications for amebic pathogenesis. Infect Immun. 2006; 74 (1): 340–51. . 16368989 10.1128/IAI.74.1.340-351.2006 PMC1346599

[8] Ehrenkaufer G, Haque R, Hackney J, Eichinger D, Singh U. Identification of developmentally regulated genes in *Entamoeba histolytica*: insights into mechanisms of stage conversion in a protozoan parasite. Cell Microbiol 2007; 9(6): 1426–44. . 17250591 10.1111/j.1462-5822.2006.00882.x

[9] Leitsch D, Radauer C, Paschinger K, Wilson I, Breiteneder H, Scheiner O, et al *Entamoeba histolytica*: Analysis of the trophozoite proteome by two-dimensional polyacrylamide gel electrophoresis. Exp Parasitol. 2005; 110 (3): 191–95. . 15955311 10.1016/j.exppara.2005.02.016

[10] Tolstrup J, Krause E, Tannich E, Bruchhaus I. Proteomic analysis of *Entamoeba histolytica*. Parasitology. 2007; 134(Pt 2): 289–98. . 17032470 10.1017/S0031182006001442

[11] McCoy J, Mann B. Proteomic analysis of Gal/GalNAc lectin-associated proteins in *Entamoeba histolytica*. Exp Parasitol. 2005; 110(3):220–25. . 15955316 10.1016/j.exppara.2005.02.013

[12] Marion S, Laurent C, Guillen M. Signalization and cytoskeleton activity through myosin IB during the early steps of phagocytosis in *Entamoeba histolytica*: a proteomic approach. Cell Microbiol. 2005; 7 (10): 1504–18. . 16153248 10.1111/j.1462-5822.2005.00573.x

[13] Okada M, Huston CD, Mann BJ, Petri WA Jr, Kita K, Nozaki T. Proteomic analysis of phagocytosis in the enteric protozoan parasite *Entamoeba histolytica*. Eukaryot Cell. 2005; 4(4): 827–31. . 15821141 10.1128/EC.4.4.827-831.2005 PMC1087816

[14] Okada M, Huston CD, Oue M, Mann BJ, Petri WA Jr, Kita K, et al Kinetics and strain variation of phagosome proteins of *Entamoeba histolytica* by proteomic analysis. Mol Biochem Parasitol. 2006; 145 (2): 171–83. . 16290089 10.1016/j.molbiopara.2005.10.001

[15] Boettner DR, Huston CD, Linford AS, Buss SN, Houpt E, Sherman NE, et al *Entamoeba histolytica* phagocytosis of human erythrocytes involves PATMK, a member of the transmembrane kinase family. PLoS Pathog. 2008; 4 (1): e8 10.1371/journal.ppat.0040008 . 18208324 PMC2211552

[16] Buss SN, Hamano S, Vidrich A, Evans C, Zhang Y, Crasta OR, et al Members of the *Entamoeba histolytica* transmembrane kinase family play non-redundant roles in growth and phagocytosis. Int J Parasitol. 2010; 40 (7): 833–43. 10.1016/j.ijpara.2009.12.007 . 20083116 PMC2866777

[17] Biller L, Matthiesen J, Kühne V, Lotter H, Handal G, Nozaki T, et al The cell surface proteome of *Entamoeba histolytica*. Mol Cell Proteomics. 2014; 13 (1): 132–44. 10.1074/mcp.M113.031393 . 24136294 PMC3879609

[18] Valdéz J, Nozaki T, Sato E, Chiba Y, Nakada-Tsukui K, Villegas-Sepúlveda N, et al Proteomic analysis of *Entamoeba histolytica* in vivo assembled pre-mRNA splicing complexes. J Proteomics. 2014; 111: 30–45. 10.1016/j.jprot.2014.07.027 . 25109466

[19] Hérnandez de la Cruz O, Muñiz-Lino M, Guillén N, Weber C, Marchant L, López-Rosas I, et al Proteomic profiling reveals that EhPC4 transcription factor induces cell migration through up-regulation of the 16-kDa actin-binding protein EhABP16 in *Entamoeba histolytica*. J Proteomics. 2014; 111: 46–58 10.1016/j.jprot.2014.03.041 . 24721673

[20] López-Rosas I, Marchant L, Gallo Olvera B, Guillén N, Weber C, Hérnandez de la Cruz O, et al Proteomic analysis identifies endoribonuclease EhL-PSP and EhRRP4 exosome protein as novel interactors of EhCAF1 deadenylase. J Proteomics. 2014; 111:59–73. 10.1016/j.jprot.2014.06.019 . 24998979

[21] Perdomo D, Aït-Ammar N, Syan S, Sachse, Jhingan GD, Guillen N. Cellular and proteomics analysis of the endomembrane system from the unicellular *Entamoeba histolytica*. J Proteomics. 2015; 112:125–40. 10.1016/j.jprot.2014.07.034 . 25109464

[22] Davis PH, Zhang X, Guo J, Townsend RR, Stanley SL Jr. Comparative proteomic analysis of two *Entamoeba histolytica* strains with different virulence phenotypes identifies peroxiredoxin as an important component of amoebic virulence. Mol Microbiol. 2006; 61 (6): 1523–32. . 16968225 10.1111/j.1365-2958.2006.05344.x

[23] David PH, Chen M, Zhang X, Clark CG, Townsend RR, Stanley SL Jr. Proteomic comparison of *Entamoeba histolytica* and *Entamoeba dispar* and the role of *E*. *histolytica* alcohol dehydrogenase 3 in virulence. PLoS Negl Trop Dis. 2009; 3 (4): e 415 10.1371/journal.pntd.0000415 . 19365541 PMC2663792

[24] Ali IK, Haque R, Siddique A, Kabir M, Sherman NE, Gray SA, et al Proteomic analysis of the cyst stage of *Entamoeba histolytica*. PLoS Negl Trop Dis. 2012; 6(5): e1643 10.1371/journal.pntd.0001643 . 22590659 PMC3348168

[25] Rengpien S, Bailey GB. Differentiaton of *Entamoeba*: a new medium and optimal conditions for axenic encystation of *E*. *invadens*. J Parasitol. 1975; 61(1):24–30. . 163898

[26] Bailey GB, Rengypian S. Osmotic stress as a factor controlling encystation of *Entamoeba invadens*. Arch Invest Med (Mex). 1980; 11(1 Suppl):11–6. . 7469635

[27] Vázquezdelara-Cisneros LG, Arroyo-Begovich A. Induction of encystation of *Entamoeba invadens* by removal of glucose from the culture medium. J Parasitol. 1984; 70 (5): 629–33. . 6512629

[28] Avron B, Stolarsky T, Chayen A, Mirelman D. Encystation of *Entamoeba invadens* IP-1 is induced by lowering the osmotic pressure and depletion of nutrients from the medium. J Protozool. 1986; 33(4):522–5. . 3795143 10.1111/j.1550-7408.1986.tb05655.x

[29] Campos-Góngora E, Viader-Salvadó JM, Martínez-Rodríguez HG, Zuñiga-Charles MA, Galindo JM, Said-Fernández S. Mg, Mn and Co Ions Enhance the Formation of *Entamoeba histolytica* Cyst-Like Structures Resistant to Sodium Dodecyl Sulfate. Arch Med Res. 2000; 31(2):162–8. . 10880721 10.1016/s0188-4409(00)00054-0

[30] Barrón-González MP, Villareal-Treviño L, Reséndez-Pérez D, Mata-Cárdenas BD, Morales-Vallarta MR. *Entamoeba histolytica*: Cyst-like structures in vitro induction. Exp Parasitol. 2008; 118(4):600–3. . 18083165 10.1016/j.exppara.2007.11.002

[31] Said-Fernández S, Campos-Góngora E, Gónzalez-Salazar F, Martínez-Rodríguez HG, Vargas-Villareal J, Viader-Salvadó JM. Mg2+, Mn2+, and Co2+ Stimulate *Entamoeba histolytica* to Produce Chitin-like Material. J Parasitol. 2001; 87(4):919–23. . 11534662 10.1645/0022-3395(2001)087[0919:MMACSE]2.0.CO;2

[32] Aguilar-Díaz H, Díaz-Gallardo M, Laclette JP, Carrero JC. *In vitro* induction of *Entamoeba histolytica* Cyst-like Structures from Trophozoites. PLoS Negl Trop Dis. 2010; 4(2):e607 10.1371/journal.pntd.0000607 . 20169067 PMC2821915

[33] Aguilar-DíazH Laclette JP, Carrero JC. Silencing of *Entamoeba histolytica* glucosamine 6-phosphate isomerase by RNA interference inhibits the formation of cyst-like structures. Biomed Res Int. 2013; 2013:758341 10.1155/2013/758341 . 23484154 PMC3581238

[34] Diamond LS, Harlow DR, Cunnick CC. A new medium for the axenic cultivation of *Entamoeba histolytica* and other *Entamoeba*. Trans R Soc Trop Med Hyg. 1978; 72(4):431–2. . 212851 10.1016/0035-9203(78)90144-x

[35] Hamzah Z, Petmitr S, Mungthin M, Leelayoova S, Chavalitshewinkoon-Petmitr P. Differential detection of *Entamoeba histolytica*, *Entamoeba dispar* and *Entamoeba moshkovskii* by a single-round PCR assay. J Clin Microbiol. 2006; 44(9):3196–200. . 16954247 10.1128/JCM.00778-06 PMC1594701

[36] Walderich B, Müller L, Bracha R, Knobloch J, Burchard GD. A new method for isolation and differentiation of native *Entamoeba histolytica and E*. *dispar* cysts from fecal samples. Parasitol Res. 1997; 83(7):719–21. . 9272565 10.1007/s004360050326

[37] Bradford MM. A rapid and sensitive method for the quantitation of microgram quantities of protein utilizing the principle of protein-dye binding. Anal Biochem. 1976; 72:248–54. . 942051 10.1016/0003-2697(76)90527-3

[38] Wisniewski J, Zougman A, Nagaraj N, Mann M. Universal sample preparation method for proteome analysis. Nature Methods 2009; 6,359–62 10.1038/nmeth.1322 . 19377485

[39] Rappsilber J, Mann M, Ishihama Y. Protocol for micro-purification, enrichment,pre-fractionation and storage of peptides for proteomics using StageTips. Nature Protocols. 2007; 2(8):1896–906 10.1038/nprot.2007.261 . 17703201

[40] Chambers M, Maclean B, Burke R, Amodei D, Ruderman D, Neumann S, et al A cross-platform toolkit for mass spectrometry and proteomics. Nature Biotechnology. 2012; 30:918–20. 10.1038/nbt.2377 . 23051804 PMC3471674

[41] Hulsen T, Vlieg J, Alkema W. BioVenn- a web application for the comparison and visualization of biological lists using area-proportional Venn diagrams. BMC Genomics 2008; 9:488 1–6 10.1186/1471-2164-9-488 . 18925949 PMC2584113

[42] Huang da W, Sherman BT, Lempicki RA. Systematic and integrative analysis of large gene lists using DAVID bioinformatics resources. Nat Protoc. 2009; 4(1): 44–57. 10.1038/nprot.2008.211 . 19131956

[43] Mi H, Muruganujan A, Thomas PD. Phanter in 2013: modeling the evolution of gene function, and other gene attributes, in the context of phylogenetic trees. Nucleic Acids Res. 2013; 4: 377–86. 10.1093/nar/gks1118 PMC3531194 23193289

[44] Mi H, Muruganujan A, Casagrande JT, Thomas PD. LArge-scale gene function analysis with the PANTHER classification system. Nat Protoc. 2013; 8:1551–66. 10.1038/nprot.2013-092 . 23868073 PMC6519453

[45] Fontana P, Cestaro A, Velasco R, Formentin E, Toppo S. Rapid annotation of anonymous sequences from genome projects using semantic similarities and a weighting scheme in gene ontology. PLoS One. 2009; 4 (2):e4619 10.1371/journal.pone.0004619 . 19247487 PMC2645684

[46] Falda M, Toppo S, Pescarolo A, Lavezzo E, Di Camillo B, Facchinetti A, et al Argot2: a large scale function prediction tool relying on semantic similarity of weighted Gene Ontology terms. BMC bioinformatics. 2012; 13(Suppl 4):S14 1-9. 10.1186/1471-2105-13-S4-S14 . 22536960 PMC3314586

[47] Radivojac P, Clark WT, Oron TR, Schnoes AM, Wittkop T, Sokolov A, et al A large.scale evaluation of computational protein function prediction. Nat Methods. 2013; 10(3):221–7. 10.1038/nmeth.2340 . 23353650 PMC3584181

[48] Yu CS, Lin CJ, Hwang JK. Predicting subcellular localization of proteins for Gram-negative bacteria by support vector machines based on n-peptide compositions. Protein Sci 2004; 13 (5):1402–06. . 15096640 10.1110/ps.03479604 PMC2286765

[49] Yu CS, Chen YC, Lu CH, Hwang JK. Prediction of protein subcellular localization. Proteins. 2006; 15; 64(3):643–51. . 16752418 10.1002/prot.21018

[50] Kolaskar AS, Tongaonkar PC. A semi-empirical method for prediction of antigenic determinants on protein antigens. FEBS Lett. 1990; 10; 276(1–2): 172–4. . 1702393 10.1016/0014-5793(90)80535-q

[51] Loftus B, Anderson I, Davies R, Alsmark UC, Samuelson J, Amedeo P, et al The genome of the protist parasite *Entamoeba histolytica*. Nature. 2005; 24; 433(7028):865–8. . 15729342 10.1038/nature03291

[52] Lorenzi HA, Puiu D, Miller JR, Brinkac LM, Amedeo P, Hall N, et al New assembly, reannotation and analysis of the *Entamoeba histolytica* genome reveal new genomic features and protein content information. PLoS Negl Trop Dis. 2010; 15:4(6): e716 10.1371/journal.pntd.0000716 . 20559563 PMC2886108

[53] Eichinger D. A role for a galactose lectin and its ligands during encystment of *Entamoeba*. J Eukaryot Microbiol. 2001; 48(1): 17–21. . 11249188 10.1111/j.1550-7408.2001.tb00411.x

[54] Ghosh SK, Van Dellen KL, Chatteriee A, Dey T, Haque R, Robbins PW, et al The Jacob2 lectin of the *Entamoeba histolytica* cyst wall binds chitin and is polymorphic. PLoS Negl Trop Dis. 2010; 20; 4(7):e750 10.1371/journal.pntd.0000750 . 20652032 PMC2907411

[55] Van Dellen KL, Chatterjee A, Ratner DM, Magnelli PE, Cipollo JF, Steffen M, et al Unique posttranslational modifications of chitin-binding lectins of *Entamoeba invadens* cyst walls. Eukaryot Cell. 2006; 5(5):836–48. . 16682461 10.1128/EC.5.5.836-848.2006 PMC1459681

[56] Husain A, Sato D, Jeelani G, Soga T, Nozaki T. Dramatic increase in glycerol byosynthesis upon oxidative stress in the anaerobic protozoan parasite *Entamoeba histolytica*. PLoS Negl Trop Dis. 2012; 6(9):e1831 10.1371/journal.pntd.000183 . 23029590 PMC3459822

[57] Lujan HD, Mowatt MR, Nash TE. Mechanisms of *Giardia lamblia* differentiation into cysts. Microbiol Mol Biol Rev. 1997; 61(3):294–304. . 9293183 10.1128/mmbr.61.3.294-304.1997 PMC232612

[58] Kliescikova J, Kulda J, Nohynkova E. Propylene glycol and contact-lens solutions containing this diol induce pseudocyst formation in *Acanthamoebae*. Exp Parasitol. 2011; 127(1):326–8. 10.1016/j.exppara.2010.08.014 . 20728440

[59] Kliescikova J, Kulda J, Nohynkova E. Stress-induced pseudocyst formation-a newly identified mechanism of protection against organic solvents in *Acanthamoebae* of the T4 genotype. Protist. 2011; 162(1):58–69. 10.1016/j.protis.2010.03.006 . 20650683

